# Mapping the path towards novel treatment strategies: a bibliometric analysis of Hashimoto’s thyroiditis research from 1990 to 2023

**DOI:** 10.3389/fendo.2023.1277739

**Published:** 2023-11-10

**Authors:** Manping Guo, Qingna Li, Xingfang Liu, Yiming Wang, Qiaoning Yang, Rui Li, Yang Zhao, Chenfei Li, Song Sheng, Hangkun Ma, Zhenghong Li, Rui Gao

**Affiliations:** ^1^ Xiyuan Hospital, China Academy of Chinese Medical Sciences, Beijing, China; ^2^ Postdoctoral Research Station, China Academy of Chinese Medical Sciences, Beijing, China; ^3^ Postdoctoral Works Station, Yabao Pharmaceutical Group Co., Ltd., Yuncheng, Shanxi, China; ^4^ Key Laboratory for Clinical Research and Evaluation of Traditional Chinese Medicine, National Medical Products Administration, Beijing, China; ^5^ National Clinical Research Center for Chinese Medicine Cardiology, Beijing, China; ^6^ Research Department, Swiss University of Traditional Chinese Medicine, Bad Zurzach, Switzerland; ^7^ Evidence Based Medicine Center, The First Affiliated Hospital of Henan University of Traditional Chinese Medicine, Zhengzhou, Henan, China

**Keywords:** Hashimoto’s thyroiditis, bibliometric analysis, autoimmune disease, papillary thyroid carcinoma, type 1 diabetes mellitus, thyroid autoimmunity, nutritional factors, treatment strategies

## Abstract

**Background:**

Hashimoto’s thyroiditis (HT), a common form of thyroid autoimmunity, is strongly associated with deteriorating clinical status and impaired quality of life. The escalating global prevalence, coupled with the complexity of disease mechanisms, necessitates a comprehensive, bibliometric analysis to elucidate the trajectory, hotspots, and future trends in HT research.

**Objective:**

This study aims to illuminate the development, hotspots, and future directions in HT research through systematic analysis of publications, institutions, authors, journals, references, and keywords. Particular emphasis is placed on novel treatment strategies for HT and its complications, highlighting the potential role of genetic profiling and immunomodulatory therapies.

**Methods:**

We retrieved 8,726 relevant documents from the Web of Science Core Collection database spanning from 1 January 1990 to 7 March 2023. Following the selection of document type, 7,624 articles were included for bibliometric analysis using CiteSpace, VOSviewer, and R software.

**Results:**

The temporal evolution of HT research is categorized into three distinct phases: exploration (1990-1999), rapid development (1999-2000), and steady growth (2000-present). Notably, the United States, China, Italy, and Japan collectively contributed over half (54.77%) of global publications. Among the top 10 research institutions, four were from Italy (4/10), followed by China (2/10) and the United States (2/10). Recent hotspots, such as the roles of gut microbiota, genetic profiling, and nutritional factors in HT management, the diagnostic dilemmas between HT and Grave’s disease, as well as the challenges in managing HT complicated by papillary thyroid carcinoma and type 1 diabetes mellitus, are discussed.

**Conclusion:**

Although North America and Europe have a considerable academic impact, institutions from emerging countries like China are demonstrating promising potential in HT research. Future studies are anticipated to delve deeper into the differential diagnosis of HT and Grave’s disease, the intricate relationship between gut microbiota and HT pathogenesis, clinical management of HT with papillary thyroid carcinoma or type 1 diabetes, and the beneficial effects of dietary modifications and micronutrients supplementation in HT. Furthermore, the advent of genetic profiling and advanced immunotherapies for managing HT offers promising avenues for future research.

## 1 Introduction

Hashimoto’s thyroiditis (HT), also known as chronic lymphocytic thyroiditis, is a form of autoimmune thyroiditis characterized by a large number of lymphocytes in its pathology (1). It is named after the Japanese surgeon Hakaru Hashimoto, who first reported four cases of the disease in 1912. Several studies have shown that HT is currently the most common autoimmune disease (2) and a major cause of endocrine disorders (3). The incidence of this disease is higher in women between the ages of 30 and 50, who are almost four times more likely to be affected by HT than men are (4). The prevalence rate in women ranges from 4.8% to 25.8%, compared with 0.9% to 7.9% in men (5), with the incidence of the condition increasing with age. The prevalence of HT varies between regions and socio-economic levels. A recent meta-analysis showed that the prevalence is highest in low- and middle-income countries, particularly in Africa (6). In addition, HT is more commonly observed in individuals diagnosed with other autoimmune diseases, such as type 1 diabetes mellitus (T1DM), Addison’s disease, systemic lupus erythematosus, or rheumatoid arthritis. These are classified under autoimmune polyglandular syndrome (7).

HT is the leading cause of primary hypothyroidism and affects a significant number of people worldwide (8). The most serious consequence of HT is the development of hypothyroidism requiring lifelong oral levothyroxine sodium tablets. Pregnant women with TPOAb-positive status are particularly susceptible to postpartum thyroiditis and potentially permanent hypothyroidism. In addition, pregnant and perinatal women diagnosed with HT have an increased risk of miscarriage, preterm birth and fetal mental retardation (9). In particular, HT creates a pro-tumorigenic microenvironment in which gene rearrangements favor the development of thyroid cancer and inflammation further contributes to cancer progression. Recent studies indicate a higher incidence of papillary thyroid cancer in HT patients compared to the average population, suggesting the role of HT as a tangible risk factor and precursor for papillary thyroid cancer (10, 11). Therefore, early detection of HT and prompt intervention are of paramount clinical importance.

The pathogenesis of HT remains unclear (7). Current scientific research broadly implicates a number of factors including autoimmunity, genetics, environmental exposures, infections and oxidative stress (7). Clinically, HT is characterized by goiter, lymphocyte infiltration and elevated serum autoimmune antibody levels. However, there is no clear evidence of a relationship between antibody titres and the risk of HT-induced (subclinical) hypothyroidism. Given the challenges in understanding the pathogenesis of HT, no definitive and effective treatments have yet been developed, and thyroid hormone replacement therapy is restricted to symptomatic or hypothyroid patients. Before the onset of hypothyroidism, the standard of care is observation, watchful waiting and regular follow-up, and medical intervention is generally unnecessary. Given these uncertainties, the etiology, diagnosis and treatment of HT warrant continued investigation. Despite the increasing number of HT-related research studies in recent years, an objective evaluation and synthesis of the research landscape are still lacking.

Bibliometrics emerged as an independent discipline in the late 1970s, integrating statistics, philology and mathematics. It has found extensive applications in document analysis (12). Using modern computer technology, bibliometric analysis can generate clear and well-visualized knowledge graphs, providing quantitative analysis methods for studying existing documents within specific fields. CiteSpace and VOSviewer are two commonly used information visualization software tools, each with its own advantages in terms of plotting. When used in combination, they can complement each other. CiteSpace uses set theory for data standardization to measure the similarity of knowledge units. It can effectively describe the knowledge evolution process and the historical time span of documents in a given cluster (13). On the other hand, VOSviewer uses a data standardization method based on the theory of probability and provides a refined and concise representation of visual knowledge maps for keywords, institutions, authors, etc. (14).

The Web of Science Core Collection (WoSCC) database is widely recognized as a high quality digital document resource, particularly suitable for bibliometric analysis, with extensive applications in fields as diverse as geology, medicine and ecology (15). This database can competently identify and summarize the state of research and emerging trends in related disciplines. The present study is based on bibliometric analysis of the WoSCC database to identify and analyze HT-related documents, with the specific objectives of (i) identifying challenging issues and research hotspots in HT; (ii) examining the research trajectory and trend in HT; (iii) constructing knowledge maps in HT; and (iv) providing valuable insights and directions for future HT research.

## 2 Materials and methods

### 2.1 Data source and retrieval strategies

**Figure 1:**
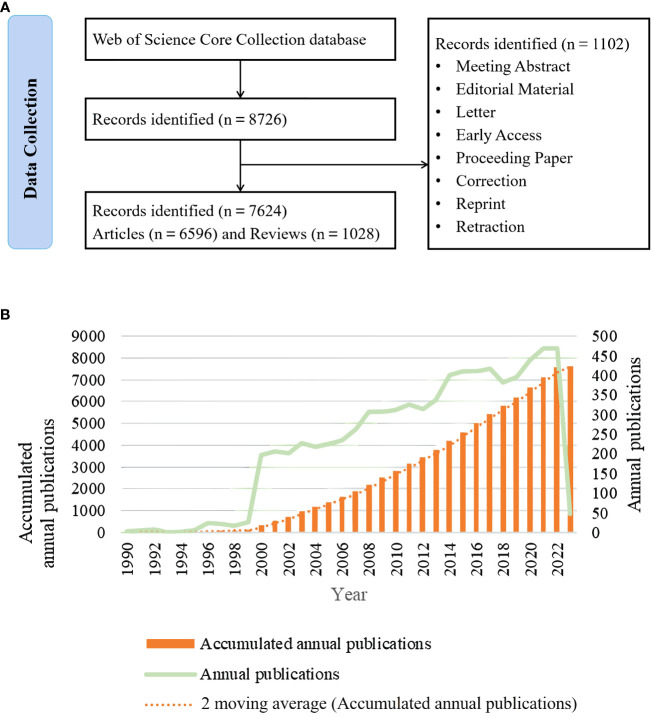
**(A)** Flowchart of literature selection; **(B)** Trends in the growth of publications worldwide from 1990 to 2023.

A comprehensive search of peer-reviewed publications on HT was conducted in the WoSCC database (https://www.webofscience.com/). The search was independently conducted by two authors (MG and YW). The main retrieval keywords included Hashimoto disease/Hashimoto’s disease, Hashimoto struma/Hashimoto’s struma, Hashimoto thyroiditis/Hashimoto’s thyroiditis, Hashimoto syndrome/Hashimoto’s syndrome, chronic lymphocytic thyroiditis, and autoimmune thyroiditis. Inclusion criteria for eligible documents were as follows: (i) publications from the WoSCC database, (ii) articles and reviews, (iii) publications from 1 January 1990 to 7 March 2023, and (iv) no language restrictions. The detailed search strategy using the WoSCC database is summarized in **Supplementary Table 1**. A total of 7,624 eligible publications (including 6,596 articles and 1,028 reviews) were included in the analysis, as shown in **Figure 1A**.

### 2.2 Data analysis

The exported data files were analyzed and visualized using CiteSpace 6.1.R3, VOSviewer 1.6.18, Bibliometrix R package and Scimago Graphica. CiteSpace 6.1.R3 was used to perform visualization analysis of institutions, references, and prediction of possible hot spot burst keywords. VOSviewer 1.6.18 was used to visualize countries, journals, authors, and cluster keywords. The Bibliometrix R Package was used to display source dynamics and topic dynamics. Scimago Graphica was used to visualize the global distribution of publications.

## 3 Results

### 3.1 Development phases and publication trends

The number of documents in a particular field and its change trend can reflect the development stage of the research field, and even evaluate and predict the development status of the research. According to the retrieval process (**Figure 1A**), 7624 publications on HT from 1 January 1990 to 7 March 2023 were included, of which 6596 articles accounted for 86.52% of the total, followed by 1028 reviews (13.48%). The number of publications and the publication time of these documents were counted, as shown in **Figure 1B**. According to their temporal characteristics, they can be divided into three phases. The first stage was the exploration stage, and although researchers explored HT between 1990 and 1999, the number of publications was relatively small; the second stage was the rapid development stage, which took place in 1999-2000, and the number of published articles increased explosively from 27 in 1999 to 196 in 2000; the third stage was the steady development stage, which began in 2000 and continues to the present. While the number of HT-related research publications has experienced fluctuations, the overall trend has shown steady growth. 2022 saw the highest number of articles published, reaching 489. However, this record number wasn’t surpassed during the entire year. However, based on the current state of research and progress, it is speculated that the field is in a stage of steady development, and there are still prospects and popularity in the future.

### 3.2 Global contributions and collaborations

**Figure 2:**
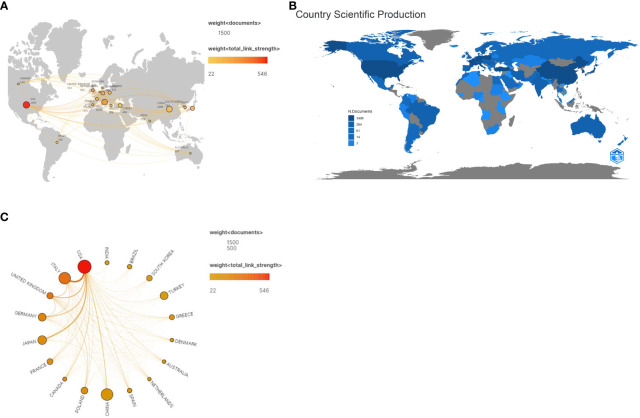
**(A)** The network map of collaboration relations between countries generated with Scimago Graphica software; **(B)** The world map of publications of countries generated with R software; **(C)** Cooperative relationships between different countries generated with Scimago Graphica software.

Over the past 33 years, HT-related research has been carried out in 98 countries/regions. Using Scimago Graphica software, we created a world map of information from published articles that could reflect the collaboration between different countries on the subject of this study (**Figure 2A**). Countries were ranked based on the number of articles published, with only the top 10 listed in **Table 1**. The United States in North America topped the list with 1405 global publications, followed by China in Asia, Italy in Europe and Japan in Asia with 1089, 1079, and 603 articles, respectively. These four countries accounted for more than half (54.77%) of global publications. Turkey in Asia and Germany in Europe also made important contributions in this field, with more than 400 articles each. Nevertheless, research in this field has been absent in some regions since 1990 (**Figure 2B**). Meanwhile, **Figure 2C** shows a map illustrating cooperation between countries in HT research.

**Table 1:** Top 10 productive countries regarding the research on Hashimoto’s thyroiditis.

Rank	Country	Documents(n)	Percentage (n/7624)	Citations	Average citations
1	USA	1405	18.43%	58390	41.56
2	China	1089	14.28%	13919	12.78
3	Italy	1079	14.15%	33557	31.10
4	Japan	603	7.91%	18365	30.46
5	Turkey	494	6.48%	6235	12.62
6	Germany	488	6.40%	15493	31.75
7	Poland	371	4.87%	5281	14.23
8	UK	314	4.12%	13854	44.12
9	France	277	3.63%	9651	34.84
10	South Korea	254	3.33%	5964	23.48

Meanwhile, a map of cooperation between countries in HT research is shown in **Figure 2C**. The results show that the United States cooperates with other countries, especially Italy, Japan, the United Kingdom, Germany, and China. Additionally, this study suggests that scientific research should not be constrained by geographical boundaries Therefore, the next step of international cooperation in conducting research in this field will be crucial.

### 3.3 Institutional contributions and collaborations

**Figure 3:**
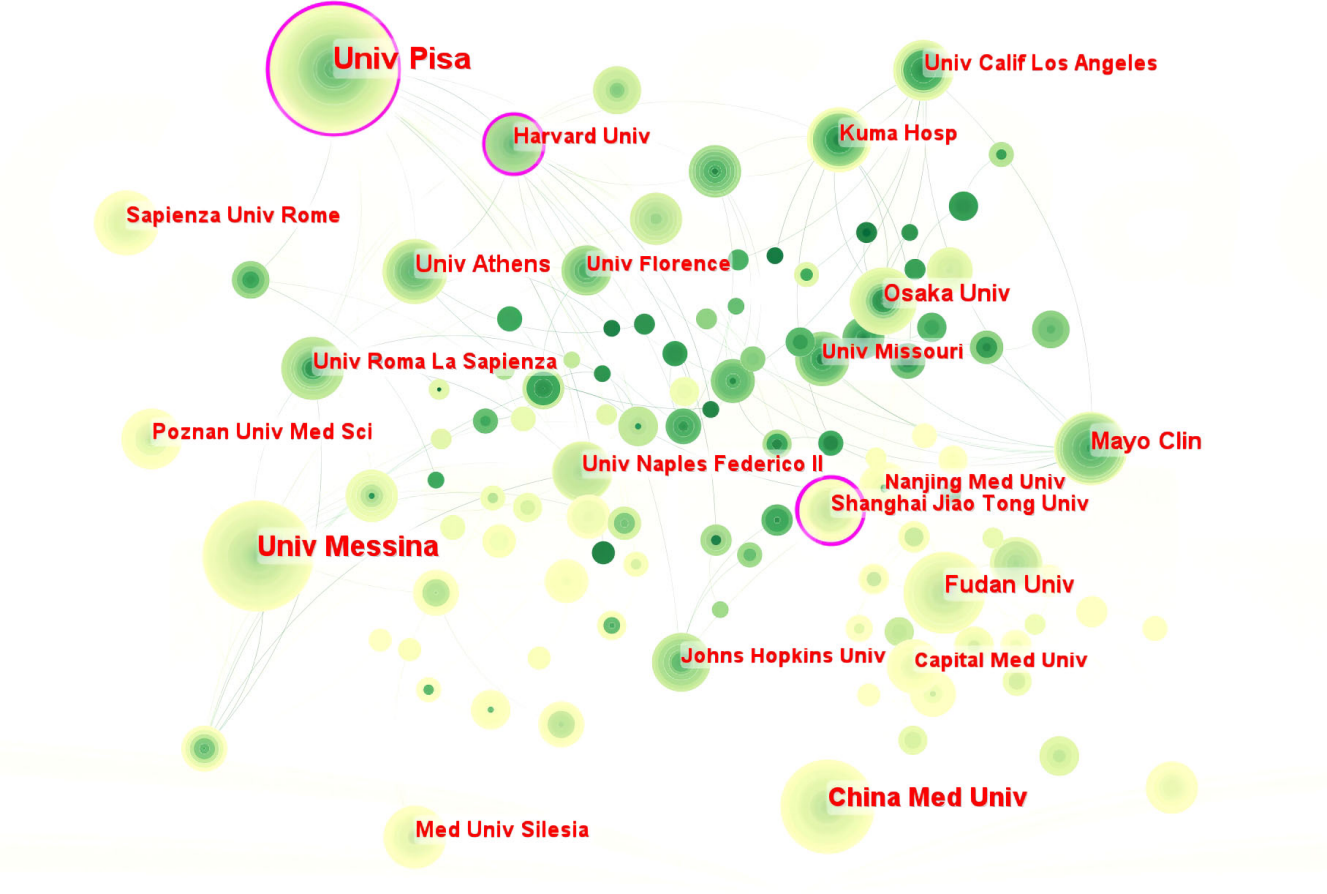
The collaboration of institutions in the field of HT.

CiteSpace was used to analyze and visualize 6181 institutions that have contributed to this field (**Figure 3**). The top 10 institutions with the highest number of publications are listed in **Table 2**. Over the last 33 years, four of the top 10 research institutions were located in Italy (4/10), including the top two, followed by China (2/10) and the United States (2/10). In terms of collaboration between institutions, we find that the institutions with a high degree of centrality (purple circle) belong to three countries: Italy, China and the United States, namely the University of Pisa, Shanghai Jiao Tong University and Harvard University. In addition, the University of Pisa ranked first with 214 publications, and the University of Messina (n = 148) and China Medical University (n = 103) also made significant contributions to HT-related research.

**Table 2:** Top 10 productive institutions regarding the research on Hashimoto’s thyroiditis.

Rank	Institution	Documents (n)	Percentage (n/7624)	Citations	Average citations
1	University of Pisa (Italy)	214	2.81%	8780	41.03
2	University of Messina (Italy)	148	1.94%	3430	23.18
3	China Medical University (China)	105	1.38%	2335	22.24
4	Fudan University (China)	88	1.15%	1544	17.55
5	Mayo Clinic (USA)	86	1.13%	3253	37.83
6	Osaka University (Japan)	79	1.04%	2114	26.76
7	University of Naples Federico II (Italy)	72	0.94%	2456	34.11
8	University of Athens (The Hellenic Republic)	68	0.89%	2197	32.31
9	University of Rome La Sapienza (Italy)	65	0.85%	2222	34.18
10	Johns Hopkins University (USA)	63	0.83%	4105	65.16

Notably, among the top 10 institutions by number of publications, Johns Hopkins University in the United States ranked tenth (n = 63), but had the highest average citation value (value = 65.16). In addition, the lack of inter-institutional collaboration is indicative of low institutional connectivity, which further underlines the importance of collaboration.

### 3.4 Leading authors and their contributions

**Figure 4:**
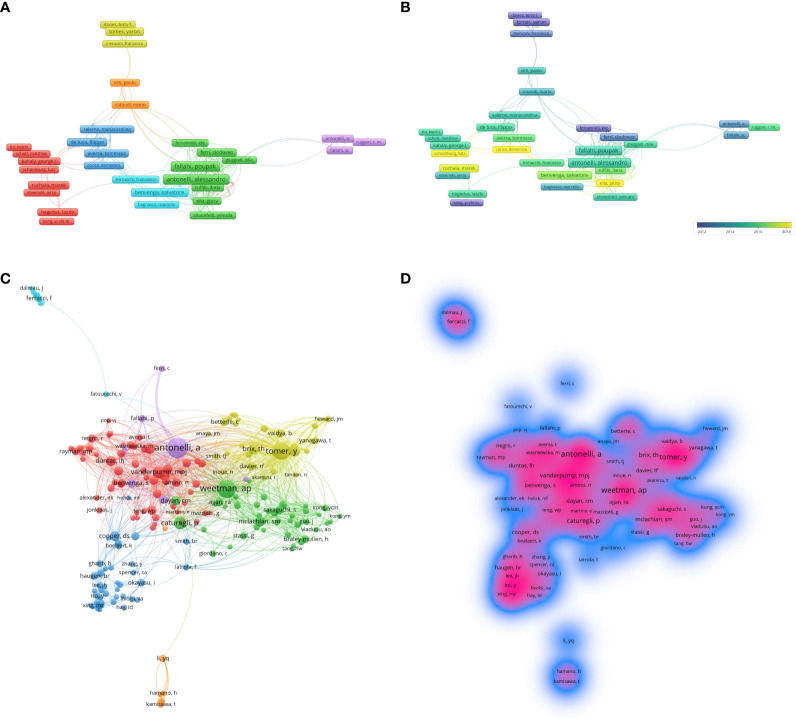
The collaboration The collaboration of authors and co-cited authors in the field of HT. **(A)** Cooperation network among the authors. **(B)** The time-overlay map of the cooperation network among the authors. **(C)** Cooperation network among the co-cited authors. **(D)** VOSviewer density visualization of the co-cited authors of institutions in the field of HT.

Over the past 33 years, a total of 33,652 authors participated in the research of HT. The top 10 most productive authors are listed in **Table 3** and include Antonelli A (n = 82), Fallahi P (n = 80), Ferrari SM (n = 72), Shan ZY (n = 58), Teng WP (n = 55), Benvenga S (n = 51), Zhang JA (n = 48), Tomer Y (n = 42), Iwatani Y (n = 33), Watanabe M (n = 33), of whom 5 are from Asia, 4 from Europe, and 1 from North America. The contribution of authors with minimal productivity of 10 publications (n=184) was visualized using VOSviewer software and shown in **Figure 4A** that their collaboration was divided into 8 clusters. When combined with the superimposed visualization of the year (**Figure 4B**), it reflects that the yellow, orange, and purple clusters were mainly active before 2016, and the clusters of other colors were still active in recent years.

**Table 3:** Top 10 productive authors in the field of Hashimoto’s thyroiditis.

Rank	Author	Institution (Country)	Documents	H-index
1	Antonelli, Alessandro	University of Pisa (Italy)	82	30
2	Fallahi, Poupak	University of Pisa (Italy)	80	29
3	Ferrari, Silvia Martina	University of Pisa (Italy)	72	28
4	Shan, Zhongyan	China Medical University (China)	58	17
5	Teng, Weiping	China Medical University (China)	55	16
6	Benvenga, Salvatore	University of Messina (Italy)	51	20
7	Zhang, Jin-an	Shanghai University of Traditional Chinese Medicine (China)	48	17
8	Tomer, Yaron	Albert Einstein College of Medicine (USA)	42	29
9	Iwatani, Yoshinori	Osaka University (Japan)	33	13

The top 10 co-cited authors are listed in **Table 4**, and the top three of them are all from the University of Pisa in Italy: Antonelli A (n = 3446), Fallahi P (n = 3409), and Ferrari SM (n = 3147). Among them, Antonelli A is the author with the most published articles and the highest average citation, indicating that he has made outstanding contributions and has a high academic impact in the field. The co-citation relationship is shown in the cluster plot (**Figure 4C**) and the influence of co-citation authors in the field is displayed in the density plot (**Figure 4D**).

**Table 4:** Top 10 productive co-cited authors in the field of Hashimoto’s thyroiditis.

Rank	Author	Institution (Country)	Citations	H-index
1	Antonelli, Alessandro	University of Pisa (Italy)	3446	30
2	Fallahi, Poupak	University of Pisa (Italy)	3409	29
3	Ferrari, Silvia Martina	University of Pisa (Italy)	3147	28
4	Tomer, Yaron	Albert Einstein College of Medicine (USA)	2584	29
5	Shan, Zhongyan	China Medical University (China)	1659	17
6	Teng, Weiping	China Medical University (China)	1612	16
7	Ferrannini, Ele	CNR Institute of Clinical Physiology (Italy)	1530	15
8	Shoenfeld, Yehuda	Ariel University (Israel)	1528	15
9	Drexhage, HA	University Medical Center Rotterdam (the Netherlands)	1523	12
10	Amino, Nobuyuki	Kuma Hospital (Japan)	1459	8

### 3.5 Journal distribution, impact, and citation analysis

**Figure 5:**
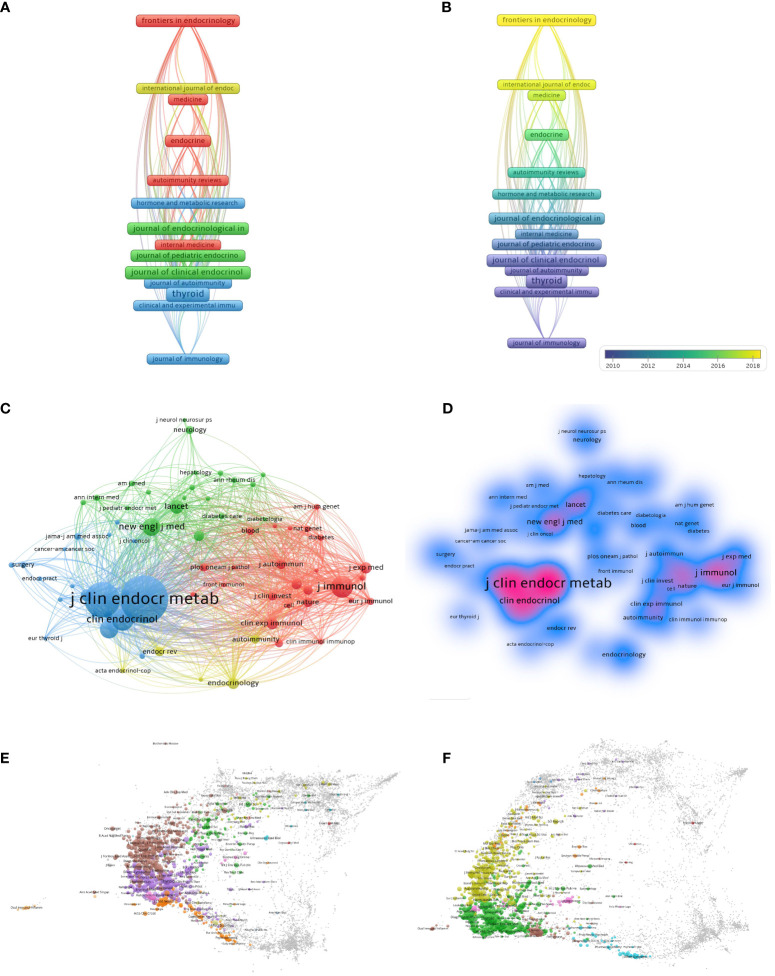
The collaboration of journals and co-cited journals in the field of HT. **(A)** Cooperation network among the journals. **(B)** The time-overlay map of the cooperation network among the journals. **(C)** Cooperation network among the co-cited journals. **(D)** VOSviewer density visualization of the co-cited journals. **(E)** The co-cited journals overlay map. **(F)** The citing journals overlay map.

A total of 1547 journals have reported on HT studies, with the top 10 publications shown in **Table 5**. *Thyroid* was the most prolific journal (n = 339, 22.06%), followed by *Journal of Clinical Endocrinology & Metabolism* (n = 202, 13.14%) and *Journal of Endocrinological Investigation* (n = 156, 10.15%). Of the top 10 journals, 4 are from the United Kingdom and 3 are from the United States. There are 5 journals with an impact factor > 5, namely *Thyroid* (6.506), *Journal of Clinical Endocrinology & Metabolism* (6.134), *Journal of Endocrinological Investigation* (5.467), *Frontiers in Endocrinology* (6.055), and *European Journal of Endocrinology* (6.558). In addition, based on the information provided by the Journal Citation Reports (JCR) evaluation system, we also found that the top 10 journals are mainly concentrated in the JCR of Q1 (40%) and Q4 (30%). The journals with minimum productivity of 40 publications (n = 21) were visualized using VOSviewer software, and as shown in **Figure 5A**, their collaboration was divided into 4 clusters. Combined with the superimposed visualization of the year (**Figure 5B**), it shows that the yellow and red clusters were mainly active after 2016, while the blue and green clusters were primarily active before 2016.

**Table 5:** Top 10 productive journals in the field of Hashimoto’s thyroiditis.

Rank	Journal	Publications (%)	Country	Impact factor (2021)	JCR
1	Thyroid	339 (22.06)	USA	6.506	Q1
2	Journal of Clinical Endocrinology & Metabolism	202 (13.14)	USA	6.134	Q1
3	Journal of Endocrinological Investigation	156 (10.15)	Italy	5.467	Q2
4	Frontiers in Endocrinology	151 (9.82)	Switzerland	6.055	Q1
5	European Journal of Endocrinology	117 (7.61)	England	6.558	Q1
6	Clinical Endocrinology	115 (7.48)	England	3.523	Q3
7	Endocrine	108 (7.03)	USA	3.925	Q3
8	Endocrine Journal	103 (6.70)	Japan	2.860	Q4
9	Journal of Pediatric Endocrinology & Metabolism	103 (6.70)	England	1.520	Q4
10	Autoimmunity	100 (6.51)	England	2.957	Q4

The influence of journals in a research field depends on the number of citations. The co-citation analysis was performed using VOSviewer (**Figure 5C**), and the density map is shown in **Figure 5D**. The size of the nodes represents the number of references, and the lines between the nodes indicate the co-citation relationship. The *Journal of Clinical Endocrinology & Metabolism*, sponsored by the United States, is the most influential journal with 18,252 citations. It is followed by the American journal, Thyroid, with 12,298 citations, demonstrating a high degree of authority in the field of HT. Among the top 10 co-cited journals, 9 journals have an impact factor of > 5, of which the impact factor of *Lancet* is 202.731, and the impact factor of *New England Journal of Medicine* is 176.082. Both Q1 and Q2 account for 40% of the journals, indicating high-quality HT-related articles and the academic significance of this study (**Table 6**). VOSviewer was combined with Pajek software to create a cited map and a citing map of the journals, as shown in **Figures 5E, F**. Limitations within VOSviewer prevented the creation of dual map overlays.

**Table 6:** Top 10 co-cited journals in the field of Hashimoto’s thyroiditis.

Rank	Journal	Citations	Country	Impact factor (2021)	JCR
1	Journal of Clinical Endocrinology & Metabolism	18252	USA	5.467	Q2
2	Thyroid	12298	USA	6.506	Q1
3	Journal of Immunology	6387	USA	5.430	Q2
4	Clinical Endocrinology	6385	England	3.523	Q3
5	European Journal of Endocrinology	5037	England	6.558	Q1
6	New England Journal of Medicine	4891	USA	176.082	Q1
7	Lancet	3460	England	202.731	Q1
8	Journal of Endocrinological Investigation	2846	Italy	5.467	Q2
9	Endocrinology	2688	USA	5.051	Q2
10	Journal of Experimental Medicine	2624	Italy	5.467	Q2

### 3.6 Key co-cited references and emerging topics

We analyzed 148,499 co-cited references and listed the top 10 references in **Table 7**. The article “ Serum TSH, T =(4), and thyroid antibodies in the United States population (1988 to 1994): National Health and Nutrition Examination Survey (NHANES III) “ published in the *Journal of Clinical Endocrinology & Metabolism* (2021 IF: 5.467) in 2002 ranked first with 438 citations. This article retrospectively analyzed the average concentrations of TSH, T4, TgAb, and TPOAb in a disease-free population (n=16533) from the NHANES III database in the United States. This population included individuals who had not reported thyroid diseases, thyroid nodules, or were not taking thyroid medication. The analysis found that approximately 18% of the disease-free population had detectable levels of TgAb or TPOAb, and a large proportion of Americans may have laboratory evidence of either hypothyroidism or hyperthyroidism without being aware of it. This finding supports the effectiveness of early screening.

**Table 7:** Top 10 co-cited references in the field of Hashimoto’s thyroiditis.

Rank	Title	Citations	Year	Journal	Type	Impact factor (2021)
1	Serum TSH, T(4), and thyroid antibodies in the United States population (1988 to 1994): National Health and Nutrition Examination Survey (NHANES III)	438	2002	Journal of Clinical Endocrinology & Metabolism	Article	5.467
2	The incidence of thyroid disorders in the community: A twenty-year follow-up of the Whickham Survey	316	1995	Clinical Endocrinology	Article	3.523
3	Chronic autoimmune thyroiditis	307	1996	New England Journal of Medicine	Review	176.082
4	Hashimoto thyroiditis: Clinical and diagnostic criteria	301	2014	Autoimmunity Reviews	Review	17.390
5	Current concepts: Thyroiditis	235	2003	New England Journal of Medicine	Review	176.082
6	Autoimmune thyroid disorders	216	2015	Autoimmunity Reviews	Review	17.390
7	Autoimmune thyroid - disease: Further developments in our understanding	212	1994	Endocrine Reviews	Review	25.261
8	2015 American Thyroid Association Management Guidelines for Adult Patients with Thyroid Nodules and Differentiated Thyroid Cancer The American Thyroid Association Guidelines Task Force on Thyroid Nodules and Differentiated Thyroid Cancer	202	2016	Thyroid	Article	6.506
9	Hashimotos disease and encephalopathy	185	1966	Lancet	Article	202.731
10	Hashimoto encephalopathy: Syndrome or myth?	184	2003	Archives of Neurology	Review	7.419

**Figure 6:**
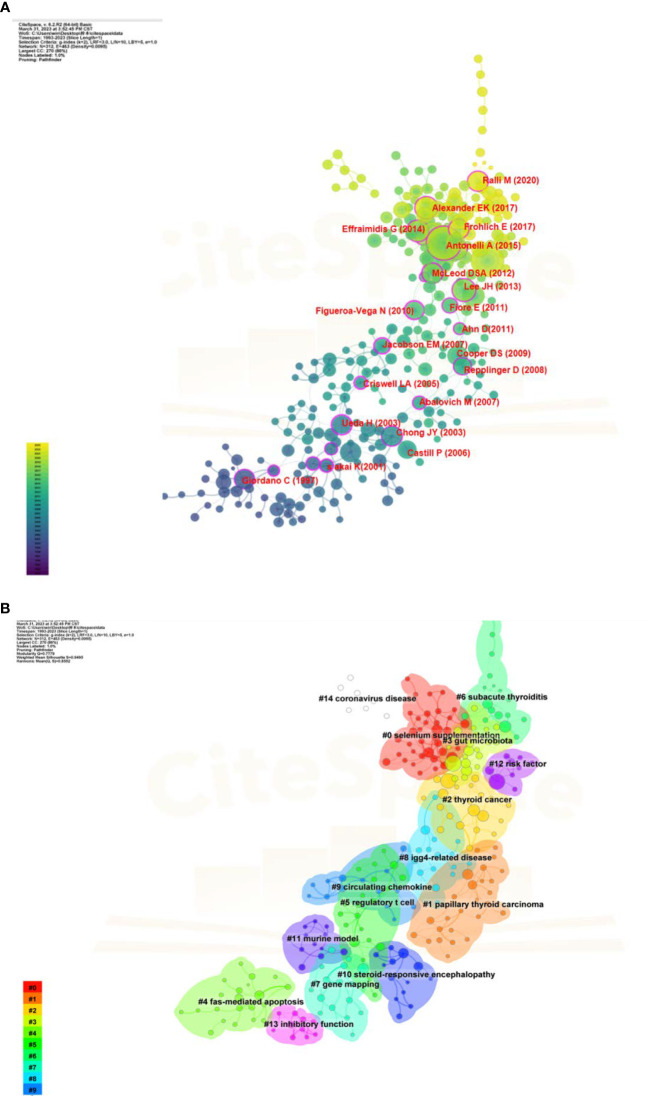
Reference analyses in the field of HT. **(A)** Co-cited networks of references. **(B)** Cluster diagrams of references.

The most recently published article was “ 2015 American Thyroid Association Management Guidelines for Adult Patients with Thyroid Nodules and Differentiated Thyroid Cancer The American Thyroid Association Guidelines Task Force on Thyroid Nodules and Differentiated Thyroid Cancer “, which published in the journal *Thyroid* (2021 IF: 6.506) in 2016. The aim of this guideline is to inform clinicians, patients, researchers, and health policy makers about the best available evidence and its limitations regarding the diagnosis and management of thyroid nodules and differentiated thyroid cancer (DTC) in adult patients. Patients with HT often have concomitant thyroid nodules, and the clinical significance of thyroid nodules lies in the need to exclude thyroid cancer. DTC consists predominantly of papillary carcinoma and follicular carcinoma, accounting for the majority of all thyroid cancers (>90%). In addition, we performed co-citation networks and clustering analysis using CiteSpace (**Figure 6**), and the references were divided into 15 clusters with active co-citations. The presence of papillary thyroid carcinoma (#1) and thyroid cancer (#2) also indicates that in HT-related studies, the differential diagnosis and disease progression of HT and thyroid cancer have always been a focus of academic attention.

Among the top 10 co-cited references, 6 are reviews and 4 are articles. Among them, the *New England Journal of Medicine* (2021 IF: 176.082) contains the most highly co-cited articles, which is one of the most prestigious peer-reviewed medical journals. This article reported the first case report of Hashimoto’s encephalopathy (HE) in 1966. Although the number of published case reports is limited so far, and the etiology of HE is not well understood, its clinical manifestations are similar to many neurological disorders. Therefore, it is often misdiagnosed, leading to speculation that the number of cases of HE is much higher than reported.

### 3.7 Keyword analysis and emerging trends

**Figure 7:**
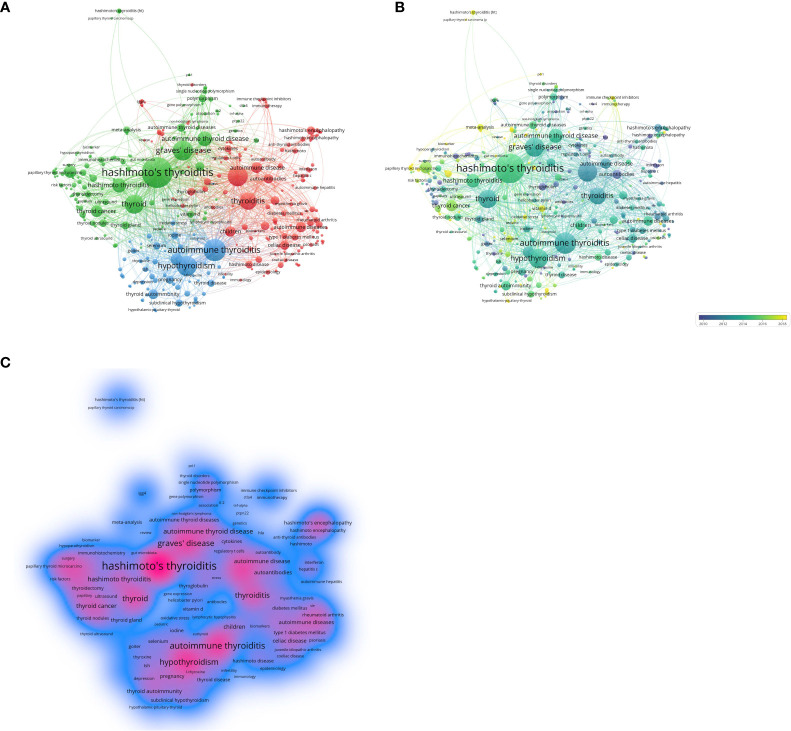
Keyword analyses in the field of HT. **(A)** VOSviewer cluster visualization of keywords. **(B)** VOSviewer density visualization of keywords. **(C)** VOSviewer overlay visualization of keywords.

Keyword analysis can reflect the current status of research topics in the HT field, including hot spots and future development directions. We constructed a co-occurrence network for high-frequency keywords and visualized them by cluster analysis (**Figure 7A**). As shown in **Supplementary Table 2**, the keywords “ hashimoto’s thyroiditis “, “ autoimmune thyroiditis “ and “ graves’ disease “ rank in the top three with 815, 742 and 608 occurrences, respectively. This suggests that the most commonly used name for HT is “ Hashimoto’s thyroiditis “. Moreover, the identification of HT and Graves’ disease in the early stage, and the mutual transformation and coexistence of the two diseases in the later stage have always been the most prominent topics in HT research. In addition, the cluster was divided into three domains according to **Figure 7A**: (i) Clinical diagnosis and identification of HT, red cluster includes “biomarkers”, “ anti-thyroid antibodies”, “ tsh receptor “, “ subacute thyroiditis “, “ thyroglobulin “ and “ addison ‘s disease “; (ii) Risk factors and treatment of HT, green cluster include “ risk factors “, “ surgery “, “ gut microbiota “, “ genetic susceptibility “, “ thyroid lymphoma “, “ vitamin d “, and “ therapy “; (iii) Complications and prevention of HT, blue cluster include “ postpartum thyroiditis “, “ breast cancer “, “ type 2 diabetes mellitus “, “ vitamin d deficiency “, “ quality of life “, “ depression “, “ selenium “ and “ iodine “. Furthermore, in **Figure 7B**, we visualized the average year of occurrence, with yellow and blue colors representing the closer and earlier keywords, respectively. In addition, we used visualization techniques to illustrate the density of the identified keywords, as shown in **Figure 7C**. The color mapping of the keywords corresponds to their relative weight, with red hues indicating higher weights.

**Figure 8:**
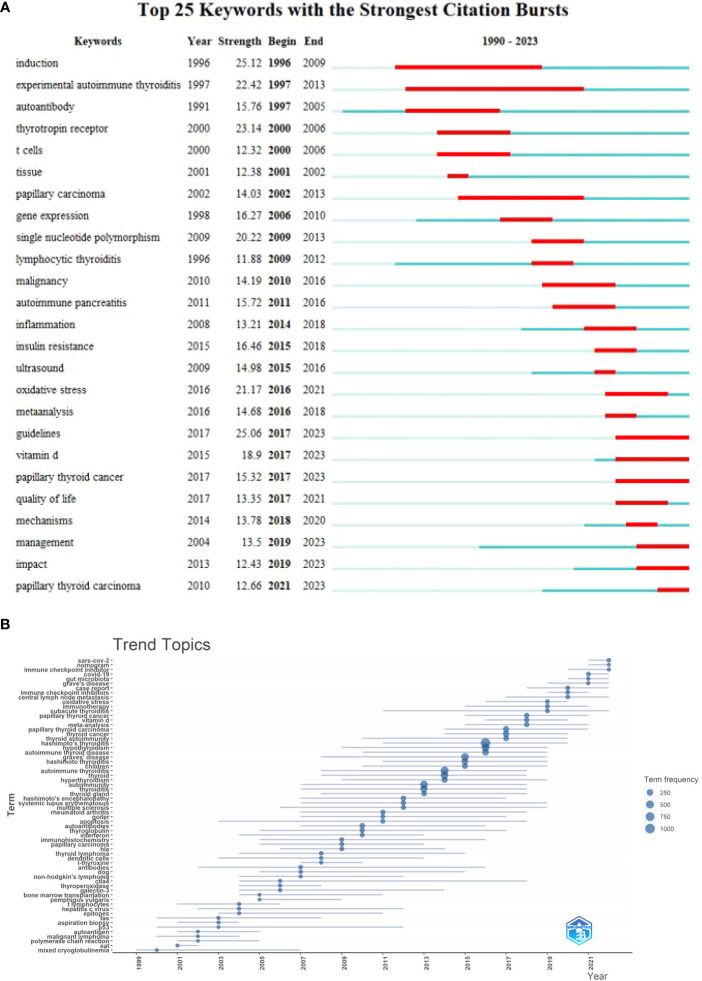
**(A)** Top 10 keywords with the strongest citation bursts; **(B)**. Topics dynamics in the field of HT generated by R software.

The “burst words” signify the emergence of keywords that have been heavily cited during a particular time period, thereby indicating the current focuses of academic inquiry. Through the use of CiteSpace software, the present study was able to identify keywords that exhibited citation bursts, resulting in the identification of 25 such keywords, shown in **Figure 8A**, with the red band indicating the burst keyword retention phase. Notably, keywords such as experimental autoimmune thyroiditis (22.42, 1997 - 2013), T cells (12.32, 2000 - 2006), gene expression (16.27, 2006 - 2010) and single nucleotide polymorphism (20.22, 2009 - 2013), showed that exploring the pathogenesis of HT through experiments has been a hot spot in HT research in the past; Keywords such as papillary thyroid carcinoma (12.66, 2021-2023), papillary thyroid cancer (15.32, 2017 - 2023), and Vitamin D (18.9, 2017 - 2023) inferred novel nutritional treatment strategies supplementing trace elements and the correlation between HT and cancer have become the focus of current research in this field. In addition, we used R software to draw the annual theme dynamic map, as shown in **Figure 8B**.

## 4 Discussion

Our research used bibliometric methods to analyze trends and hotspots in HT-related research by examining publication output, growth of research interest, leading countries/regions, international collaborations, top institutions, preferred journals, and author keywords.

### 4.1 Research phases of development and influencing factors

By analyzing the annual production of publications, we identified three phases in the development of the HT field over the past decades: the initial exploration stage, the rapid development phase, and the steady development stage. The number of publications in the rapid development stage increased explosively from 27 in 1999 to 196 in 2000, and this change in development speed may be attributed to the improvement of HT detection methods, the in-depth study of pathogenesis, and the development of clinical trials. Improvements in examination methods have been seen, such as the color Doppler measurement of inferior thyroid arterial blood flow in patients with autoimmune thyroid disease (AITD) (16), fine-needle aspiration of thyroid nodules (17), and the emergence of novel TSH receptor antibody assays (18). In addition, advances in the genetics, cytology, and histology of Hashimoto’s disease have paved the way for a more thorough investigation of the pathogenesis (19, 20). Case reports (21) and observational clinical trials (22) have revealed the current state of research on the complex symptoms, multiple complications, and treatment limitations of Hashimoto’s disease. It is noteworthy that the expansion of annual publications has plateaued since the previous year, indicating that the progress of HT has been impeded. In particular, the lack of effective remedies for HT remains a concern, and in cases where HT develops into hypothyroidism, the majority of patients require lifelong thyroid hormone replacement. Notably, the manifestation of clinical symptoms persists in many patients even after normalization of thyroid hormone replacement (23).

### 4.2 Dissecting the imbalance of academic resources

In terms of collaborative networks of countries, institutions, and authors, a persistent problem is the chronic imbalance in academic resources between developing and developed countries. For example, China and Turkey, as representatives of developing countries, have already been among the top countries in the world in terms of the number of publications, ranking second and fourth respectively, but their influence towards other countries in this field remains weak, as reflected in the fact that the average citations of the two countries rank first and second last among the top 10 countries in terms of the number of publications. Among the top 10 institutions for publications, 8 are located in developed countries, four of which are located in Italy. Only 2 institutions from the developing world, Fudan University and China Medical University, are in China. Notably, these two Chinese institutions rank near the bottom in terms of average citations among the top 10. This phenomenon can be explained in two ways. First, with rapidly expanding economies, scientific research has developed rapidly in growing countries. However, compared to developed countries, many of their scientific studies are characterized by replication and imitation. Second, economically developed countries and universities with higher academic status have more research resources and financial support, which encourages innovative practices. In addition, these institutions enjoy more opportunities for international exchange, which results in a notable academic gap between developed and developing regions. Nevertheless, Shanghai Jiaotong University, a Chinese research institution, has established initial partnerships with research institutions from other countries in recent years.

### 4.3 Journal influence and selection considerations

According to the comprehensive results of the number of publications and citations, *Thyroid* and *Journal of Clinical Endocrinology & Metabolism* are the most influential journals in this field. *Thyroid* is the official journal of the American Thyroid Association (ATA), the only thyroid journal included in SCI, and is the most authoritative journal in the field of thyroid diseases, publishing articles covering clinical and experimental articles in the field of thyroid research. *Journal of Clinical Endocrinology & Metabolism* is also a top-level journal in the field of endocrinology. It is worth noting that the comprehensive journals *New England Journal of Medicine* (IF: 176.082) and *Lancet* (IF: 202.731) are not in the top ten in terms of the number of publications, but the number of citations ranks sixth and seventh, indicating that the quality of the publications of these two top journals is high. Analysis of journals can help researchers choose the right journals to submit papers, prioritizing high-impact journals while also considering the journal’s ability to disseminate research to the right audience and contribute to the development of the field.

### 4.4 Research hotspots

Based on the results of keywords clustering and citations analysis, the following suggestions are provided for the future research hotspots in the field of HT.

#### 4.4.1 Coexistence and diagnostic challenges of graves’ disease in HT

The keyword “graves’ disease” ranked first three with 608 occurrences, indicating that it often occurs at the same time as HT (24). Graves’ disease (GD) and HT are the most common types of AITD (25). In **Table 7**, the article “Chronic autoimmune thyroiditis” published in the New England Journal of Medicine ranks 3rd in terms of citation frequency which extensively discusses the similarities and differences between HT and GD. GD is mainly manifested as hypermetabolic syndrome caused by hyperthyroidism, with diffuse enlargement of the thyroid gland and positive expression of TRAb. Approximately 70% of GD patients have positive TPOAb and TGAb expression (26). However, during the early stages of HT, clinical manifestations of hyperthyroidism, positive TGAb and TPOAb expression, and positive TRAb expression can also be observed in a small number of patients (27). Case studies have also shown that HT can progress to GD (28, 29). In addition, approximately 10-15% of GD patients may develop hypothyroidism following antithyroid therapy (30), strongly suggesting the coexistence of GD and HT. Thus, it is difficult for clinicians to differentiate between GD and HT, especially when presenting with atypical clinical symptoms and positive antibody test results in the early stages of either disease. Current diagnostic criteria remain unclear and can lead to serious adverse outcomes if incorrect treatment plans are pursued. Therefore, it is imperative that future research focus on developing clear and objective diagnostic criteria to ensure that patients receive the most effective and clinically appropriate treatments.

#### 4.4.2 Gut microbiota and its implications in HT

As shown in **Figure 8B**, “gut microbiota” has emerged as a hot keyword over the past three years. Recent studies have shown that the gut microbiota may play an important role in the induction of HT (31). This study conducted co-citation network and clustering analysis of the co-cited references in HT-related research. It was found that gut microbiota (#3) is one of the clusters with active co-citation relationships. Several studies support the role of the gut commensal microbiota in autoimmune genesis (32, 33), but the role in HT has not received attention until recent years. Lerner A et al. (34) first proposed the concept of the “gut-thyroid axis” in their research. In the body’s internal environment, only a single layer of epithelial cells separates luminal contents from effector immune cells, and disruption of the epithelium drives autoimmunity (32). 40% of HT patients suffer from lymphocytic colitis with elevated intraepithelial lymphocyte counts, further implicating the role of gut microbiota (35). Microbial metabolites have been shown to affect thyroid function and activate the immune system (36–38). Lipopolysaccharide-induced Toll-like receptor activation is associated with the development of thyroiditis or the production of anti-thyroglobulin antibodies in mice (39). Changes in the gut microbiota are clearly associated with the pathogenesis of HT, but their role in the induction or protection of thyroid autoimmunity remains to be investigated.

#### 4.4.3 Complications and challenges: PTC and T1DM

As shown in **Figure 8A**, “papillary thyroid carcinoma”(12.66, 2021 - 2023) has become a prominent term in the last 3 years. Furthermore, papillary thyroid carcinoma (#1) and thyroid cancer (#2) were also identified as two important clusters with active co-citation relationships in this study. Since 1955, when Dailey et al. (40) first reported the association between HT and papillary thyroid cancer (PTC), the relationship between the two diseases has been the focus of research and controversy. Despite the controversy, many *in vitro* and *in vivo* studies have suggested that thyroid autoimmunity and thyroid cancer, mainly PTC, may coexist (41, 42). The inflammatory process of HT is considered a potential risk factor for the development of thyroid cancer (43). Lymphocytic infiltration of HT is common in the thyroid gland that has been partially removed due to tumor (44). Several recent studies have reported an increased incidence of PTC in HT patients after thyroidectomy, especially in women (45–48). On the other hand, the incidence of primary thyroid lymphoma is significantly increased in HT patients, strongly suggesting a pathogenesis link between this autoimmune disease and malignant thyroid lymphoma (49). In the future, it will be necessary to understand the relationship between thyroid autoimmunity and cancer and to develop tailored treatment options for PTC patients with AITD.

As shown in **Table 7**, the number of occurrences of the keyword “type 1 diabetes” ranks in the top 30. Comparatively, individuals with T1DM have an increased susceptibility to other autoimmune diseases compared to the general population, with this risk increasing over the past two decades (50). To further underscore such concerns, patients diagnosed with T1DM exhibit a greater incidence of thyroid dysfunction in contrast to both type 2 diabetes and non-diabetic controls. Specifically, patients with T1DM have an incidence of autoimmune thyroiditis ranging from 3 to 50 percent (51). A population-based twin study has provided compelling evidence of etiologic overlap between T1DM and HT, suggesting that both disorders may share an identical origin (52). Similar to hyperthyroidism, hypothyroidism appears to affect glycemia in patients with T1DM (53), thereby increasing the risks of both diabetic retinopathy and diabetic nephropathy (54). Indeed, the clinical management of patients with both T1DM and HT poses significant challenges.

#### 4.4.4 Nutritional factors in HTs: current understanding and controversies

As shown in **Figure 8A**, vitamin D (18.9, 2017 - 2023) is a burst word that has been active in the last 5 years. “Selenium” is also among one of the top 30 keywords in terms of frequency. Furthermore, the co-citation network and cluster analysis results of the co-citation references showed that selenium supplementation (#0) is one of the clusters with active co-citation relationships in this study. Nutritional deficiencies often accompany HT patients, and an appropriate diet with appropriate supplementation may be a critical aspect of the treatment process (55). However, the effects of selenium, vitamin D, iodine, iron, magnesium, and vitamin B12 on HT remain controversial. While some studies suggest a beneficial effect of vitamin D supplementation in HT patients by reducing thyroperoxidase antibody titers (56, 57), most studies show no significant associations between serum vitamin D and thyroglobulin antibody, thyroid-stimulating hormone, free triiodothyronine, or free thyroxine concentrations (5). In addition, excessive iodine intake has been shown to increase the incidence of HT (4). Further research is needed to evaluate the relationship between HT and selenium, magnesium, iron, and vitamin B12 levels. Although there are no specific dietary recommendations for patients with HT, studies support that a Mediterranean diet, rich in omega-3 fatty acids found in fatty fish (58), and an anti-inflammatory diet containing vitamins, minerals, and polyphenols may provide protective effects (59, 60). Evidence suggests that a gluten-free diet may also be beneficial for people with HT (61, 62), but further studies are needed. Medical therapy, in conjunction with appropriate diet and supplementation, is an important aspect of medical care for patients with HT. Therefore, more research is needed to determine the role of micronutrients and diet in the development and progression of HT.

### 4.5 Limitations

The analysis is subject to certain limitations. First, we only included English articles from the Web of Science database, possibly causing an over-representation of English-speaking countries’ research. Second, taking citation count as a major factor would mean that very recent work although influential might not get featured in the study, and citation counts as an impact measure don’t necessarily reflect the quality of research.

Moreover, the keyword analysis depends on the indexed keywords, potentially leading to inaccuracies in topic representation. Also, the study only covers articles published until 2023, and the research landscape may evolve with time. In addition, the software is currently unable to match and output countries with their respective published articles, which may result in the inability to conduct a comparative analysis of the quantity and types of research articles, as well as the specific mechanisms they focus on across different countries. It also hinders the exploration of potential links between variations in population proportions among different countries and differences in prevalence rates of HT. Despite these limitations, this study provides a comprehensive review of HT research and identifies key trends for future studies.

## 5 Conclusion

This bibliometric study has thoroughly investigated the landscape of HT research, illuminating significant trends, key contributors, and emergent hotspots. Although North America and Europe currently exert substantial influence in the academic domain, institutions in emerging nations like China are exhibiting increasing momentum in HT studies.

Research hotspots encompass areas such as the challenge of differentiating HT and Graves’ disease, the complex link between gut microbiota and HT onset, and the management of HT alongside complications like papillary thyroid carcinoma or type 1 diabetes. The impact of dietary modifications and micronutrient supplementation on HT treatment is also a burgeoning research focus.

Furthermore, the emergence of sophisticated genetic profiling techniques and advanced immunotherapies brings a wave of exciting new possibilities for HT management. These trends mark out promising future research trajectories, paving the way for more comprehensive understanding and effective treatment strategies for HT. The information gleaned from this study serves as a springboard for researchers aiming to make impactful contributions to the field of HT research.

## Data availability statement

The raw data supporting the conclusions of this article will be made available by the authors, without undue reservation.

## Author contributions

MG: Conceptualization, Visualization, Writing – original draft. QL: Data curation, Writing – review & editing. XL: Supervision, Validation, Writing – review & editing. YW: Methodology, Writing – original draft. QY: Methodology, Writing – original draft. RL: Methodology, Writing – original draft. YZ: Formal Analysis, Writing – original draft. CL: Formal Analysis, Writing – original draft. SS: Software, Writing – original draft. HM: Software, Writing – original draft. ZL: Conceptualization, Writing – original draft. RG: Writing – review & editing, Conceptualization.
