# Supplementary material for: Mapping the path towards novel treatment strategies: a bibliometric analysis of Hashimoto’s thyroiditis research from 1990 to 2023
Source: Front Endocrinol (Lausanne). 2023 Nov 10;14:1277739. doi: 10.3389/fendo.2023.1277739 (PMC10667915; doi:10.3389/fendo.2023.1277739)
Supplement: Supplementary file 1 [file Table_1.docx]

**SUPPLEMENTARY TABLE 1. Retrieval strategy of HT in the WoSCC**

| Items | Inclusion Criteria |
| --- | --- |
| Research database | WoSCC |
| Retrieval keywords | Hashimoto disease / Hashimoto’s disease  Hashimoto struma / Hashimoto’s struma  Hashimoto thyroiditis / Hashimoto’s thyroiditis  Hashimoto syndrome / Hashimoto’s syndrome  chronic lymphocytic thyroiditis  autoimmune thyroiditis |
| Language | No limit |
| Type of articles | Articles and Reviews |
| Searching period | January 1,1990 to March 7, 2023 |
| Data collection | Export with full records and cite reference in plain text format |
| Sample size | 7624 publications including 6596 articles and 1028 reviews. |

**SUPPLEMENTARY TABLE 2. The top 30 hotspots keywords with the highest frequencies.**

| **Rank** | **Keywords** | **Occurrences** | **Rank** | **Keywords** | **Occurrences** |
| --- | --- | --- | --- | --- | --- |
| 1 | hashimoto's thyroiditis | 980 | 16 | thyroid autoimmunity | 129 |
| 2 | autoimmune thyroiditis | 566 | 17 | autoantibodies | 124 |
| 3 | graves' disease | 465 | 18 | celiac disease | 116 |
| 4 | hypothyroidism | 463 | 19 | hashimoto's disease | 105 |
| 5 | autoimmunity | 461 | 20 | autoimmune | 98 |
| 6 | thyroid | 421 | 21 | type 1 diabetes | 98 |
| 7 | thyroiditis | 360 | 22 | autoimmune thyroid diseases | 97 |
| 8 | autoimmune thyroid disease | 273 | 23 | hashimoto's encephalopathy | 92 |
| 9 | hashimoto thyroiditis | 253 | 24 | pregnancy | 90 |
| 10 | papillary thyroid carcinoma | 199 | 25 | thyroid gland | 90 |
| 11 | thyroid cancer | 180 | 26 | hashimoto disease | 82 |
| 12 | hyperthyroidism | 980 | 28 | thyroglobulin | 81 |
| 13 | autoimmune disease | 566 | 27 | polymorphism | 81 |
| 14 | children | 465 | 29 | ultrasonography | 80 |
| 15 | autoimmune diseases | 463 | 30 | selenium | 77 |
